# Supplementary material for: Patterns of Intron Gain and Loss in Fungi
Source: PLoS Biol. 2004 Nov 30;2(12):e422. doi: 10.1371/journal.pbio.0020422 (PMC532390; doi:10.1371/journal.pbio.0020422)
Supplement: Table S1 — Also available at http://genes.mit.edu/NielsenEtAl/. (4.3 MB ZIP). [file pbio.0020422.st001.zip › NielsenEtAl/html/1264.html]

AN1117.1.NCU03319.1.MG09309.1.FG00538.1


```
 CLUSTAL W (1.82) Multiple Sequence Alignments - Introns Inserted


Sequence 1: NCU03319.1	302 aa
Sequence 2: MG09309.1	302 aa
Sequence 3: FG00538.1	302 aa
Sequence 4: AN1117.1	303 aa
Alignment Length: 304 aa
Number Identitical Residues: 193 aa
Alignment Score (without introns) 8790


MG09309.1 	MSQ-RGPSGYGIPNAPNPFGGPSSQPDGQG-SALDAIREQTSKIEDMLDSVSEPIKP2YL
NCU03319.1	MAQMRGPGSYGIG-ASSPFGGPVNEDSTES-SPLDAIRKQTSKIEDVLDSFSEPIKP2YL
FG00538.1 	MSN-RFNSGYGLG-GPAPYGGSQAAEPQQGEGFLEQIRPYTSKIEDALDTISEPVKP2YL
AN1117.1  	MAQIRGTAGYNLG-HQNPFGGPGSAAATNDPSPLDTIREHTSKIEDWLDTISDPIKP2YL
          	*:: *  ..*.:     *:**.      :. . *: **  ****** **:.*:*:** **

MG09309.1 	PAIGRFLIVVTFIEDALRILTQWSDQVYYLRDYRGF1PRGFPQLFLIINVVAMVVCSGMV
NCU03319.1	PAIGRFLIVVTFIEDALRIMTQWNDQLLYLHDYRHI1PNGVTHLFLLVNVIAMASCSTLV
FG00538.1 	PAIGRFLIVVTFFEDALRIVTQWSDQLLYLKDYRHI1PSGITHLFLLVNIIAMFSCSTLV
AN1117.1  	PAVGRFLIVVTFLEDSLRILTQWSDQLVYLRDYRKI1PWGITHAFLILNVITMTICSLLV
          	**:*********:**:***:***.**: **:*** : * *..: **::*:::*  ** :*

MG09309.1 	ITRRFSEYAVGGLIAVVITQALGYGLIFDLNFFLRNLSVMGGLIMVLSDSWVRKTVNFAG
NCU03319.1	IIRKYSEYAVGGLIGVVITQALGYGLIFDLNFFLRNLSVMGGLLMVLSDSWVRKTKAFAG
FG00538.1 	IIRKHSDYAVAGLMGVVVTQALGYGLIFDLNFFLRNLSVIGGLVMVLSDSWVRKTQVFAG
AN1117.1  	IGRKHAEFAVAGLLGVVVVQGLGYGLIFDLNFFLRNLSVVGGLLMVLSDSWVRKRFVPAG
          	* *:.:::**.**:.**:.*.******************:***:**********    **

MG09309.1 	LPQLEEKDRKMYFQLAGRVLLIFLFIGFVFAGEWSIWRVMVSIVGLVACIMVVVGFKAKF
NCU03319.1	LPTLEEKDRKMYFQLAGRVLLIFLFIGFVFSGEWTIWRIIVSLVGLVACVMVVVGFKTKF
FG00538.1 	LPQIDEKDRKMYFQLAGRVLLIFLFIGFVFSGEWSLWRVIVSSLGGISCVMVVVGFKAKY
AN1117.1  	LPQLDEKDRKMYVQFAGRVLLIFLFIGFIFSGQWSLWRVVVSLFGFVACVMVIVGFKAKF
          	** ::*******.*:*************:*:*:*::**::** .* ::*:**:****:*:

MG09309.1 	SATLLVVILSIFNLFVNNFWT0LHEHHPHKDFAKYDFFQILSIV~GGLLLLVNSGPGQFS
NCU03319.1	SSTLLVVILSIFNLLVNNFWT0LHEHHPHKDFAKYDFFQILSIV~GGLLLLTNTGPGQFS
FG00538.1 	SATLLVVILSIFNLLVNNFWT0LHEHHPHKDFAKYDFFQILSIV~GGLLLLVNSGPGQFS
AN1117.1  	SAILLVLLLSVFNILVNNFWT0LHPHHPHKDFAKYDFFQILSIV2GGLLLLVNMGPGQLS
          	*: ***::**:**::****** ** ******************* ******.* ****:*

MG09309.1 	IDEKKKVY
NCU03319.1	IDEKKKVY
FG00538.1 	IDEKKKVY
AN1117.1  	MDEKKKVY
          	:*******
```
